# Drought Adaptation and Responses of *Stipa krylovii* Vary Among Different Regions: Evidence From Growth, Physiology, and RNA‐Seq Transcriptome Analysis

**DOI:** 10.1002/ece3.70870

**Published:** 2025-01-21

**Authors:** Ziqing Gong, Zehang Qu, Yulin Liu, Tao Wang, Baijie Fan, Anzhi Ren, Yubao Gao, Nianxi Zhao

**Affiliations:** ^1^ Department of Plant Biology and Ecology, College of Life Science Nankai University Tianjin P. R. China

**Keywords:** differentially expressed genes (DEGs), drought response, drought stress, RNA‐seq, *Stipa krylovii*

## Abstract

In the context of global climate change, exploring how plant adaptation and responses to drought vary among different regions are crucial to understanding and predicting its geographic distribution. In this study, to explore the drought adaptation and responses of the dominant species in the semi‐arid Eurasian Steppes and their differences among the different regions in terms of growth, physiology, and RNA‐seq transcriptome, *Stipa krylovii* was chosen as the study material, and a seed source (three regions: eastern, middle, and western regions) × soil moisture treatment (three treatments: control, light drought, and heavy drought) two‐factor experiment was conducted. (1) Four growth traits for individuals from the western region were significantly lower than those from the other two regions. By Kyoto Encyclopedia of Genes and Genomes (KEGG) functional enrichment analysis on gene expressions of individuals from each treatment, unique enriched pathways were found under heavy drought. (2) The decrease in the number of tillers with the increasing drought was much lower for individuals from the western region than those from the other two regions. The differentially expressed genes (DEGs) of individuals from the eastern, middle, and western regions between heavy drought versus control were 4887, 1900, and 4896. By KEGG functional enrichment analysis, individuals from the eastern and middle regions mainly regulated energy metabolism and metabolism of other amino acids; and those from the western region mainly regulated biosynthesis of other secondary metabolites and carbohydrate metabolism. (3) Clustering analysis based on gene expressions separated the western region from the other two regions under the same drought treatment. This study indicates that drought adaptation and responses of *S. krylovii* vary among different regions, especially between individuals from the western region and the other two regions. These findings are essential to understanding the adaptive evolution of population and germplasm resource protection for this important species.

## Graphical abstract

Drought tolerance of *Stipa krylovii* is greater in the western region. Individuals from the eastern and middle regions show similar drought adaptation mechanisms. *S. krylovii* regulates metabolic pathways to respond to heavy drought.

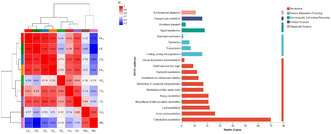

## 1 Introduction

Since the 20th century, droughts in arid and semiarid regions have been occurring more frequently and extensively as a result of global climate change (Mukarram et al. 2021). In the natural environment, terrestrial plants can change their morphology, structure, physiological, and biochemical characteristics and even the level of genetic material in response to changes in ecological factors, increasingly molding their adaptation and response mechanisms by long natural selection (McIntyre et al. 1999). In other words, the drought adaptation and response mechanisms of plants show great variance and diversity among or within species (de Dorlodot et al. 2007); however, they are influenced by both genetic differentiation (local adaptation) and environmental modification (phenotypic plasticity) and are related to seed sources (Klein and Mitchell 2024). Therefore, exploring how individuals from different regions of a key plant species adapt and respond to drought and estimating their differences and diversity are essential to understanding and predicting population evolution potential and distribution region shift.

Plant growth, morphology, physiological, and behavior traits, and DNA‐based molecular methods have been proven powerful to explore the differences in drought adaptation and response mechanisms among populations or regions of a species (Pacini and Dolferus 2019; Qian et al. 2021; Seiler and Cazell 1990; Wang et al. 2006). However, these methods are limited in explaining how they regulate plant physiological functioning. In the past years, RNA‐seq technology has been widely used to study how different samples within a species regulate plant physiological functioning to adapt and respond to environmental stresses in the absence of information on the whole genome sequence (Jin et al. 2013; Villarino et al. 2014; Zou et al. 2022). For RNA‐seq data of the plant samples from different environmental treatments, differentially expressed genes (DEGs) between treatments could be found, and these DEGs can be used for Kyoto Encyclopedia of Genes and Genomes (KEGG) functional enrichment and/or annotation analysis to explore how they regulate the response processes in the face of environmental changes and consequently to compare response differences between or among different samples. For example, Nawae et al. (2020) have analyzed the DEGs between two water conditions for two genotypes of sugarcane, KPS01‐12 (drought‐tolerant genotype) and UT12 (drought‐sensitive genotype), and found that it is KPS01‐12 but not UT12 that upregulates expression of genes related to drought tolerance such as water retention and antioxidant secondary metabolite biosynthesis. Precipitation pattern changes and an increase in the frequency of extreme droughts in globally sensitive regions have resulted in increased habitat patches and shifts in the distribution region of some species (Abreha et al. 2022), which have caused significant population differences owing to genetic drift and reduced gene flow. Therefore, in order to better understand and predict community dynamics and species distribution area fluctuations, more researches are needed to use RNA‐seq to analyze the drought adaptation and response mechanisms of the dominant species in these sensitive important regions (Ma et al. 2018).

As an important part of the arid and semiarid Eurasian Steppe, climatic characteristics of the Inner Mongolia Steppe show decreasing precipitation and increasing annual mean temperature from northeast to southwest, which provides an ideal area for studying drought adaptive differences of a certain species by sampling from different regions in the sensitive region. *Stipa krylovii* is one of the important dominant species in the Inner Mongolia Steppe; its morphological and physiological traits and trait responses show significant differences among populations/regions caused by genetic differentiation and environmental modification (Gu et al. 2015; Wang et al. 2006; Zhao et al. 2016). To our knowledge, no study has explored the difference in adaptation strategies and the diversity of response processes to drought among different *S. krylovii* regions from the aspect of RNA‐seq transcriptome. The gaps of knowledge would limit us to further understanding the drought adaptation difference and response mechanisms diversity of *S. krylovii* as well as population evolution and its distribution region changes.

In the present study, seedlings of *S. krylovii* from three regions (eastern, middle, and western regions; three populations per region) were used as plant materials and a seed source of region (eastern, middle, and western regions) × soil moisture treatment (control, light‐drought, and heavy‐drought treatments) two‐factor experiment was carried out under the controlled conditions to explore the performance of *S. krylovii* under each treatment in the aspect of growth (number of tillers, plant height, aboveground biomass, and belowground biomass), physiological (net photosynthetic rate, transpiration rate, and stomatal conductance) traits, and RNA‐seq‐based transcriptome. Meanwhile, gene expressions and DEGs between soil moisture treatments versus control for *S. krylovii* from each region were analyzed by KEGG functional enrichment analysis. Specifically, we proposed the drought adaptation and response mechanisms of *S. krylovii* were dependent on its seed source of regions, and such strategies of individuals from the eastern and middle regions were relatively similar and were different from the western region, considering that the distribution regions of *S. krylovii* increased in the past decades and the western part of the distribution area is more arid and unfertile than the central and eastern parts (Liu 2004).

## 2 Materials and Methods

### 2.1 Experimental Materials

In the primary distribution zones of *S. krylovii* , spanning nine distinct populations across three diverse regions (the eastern, middle, and western regions) along the annual precipitation gradient, seeds were collected by population and the climatic characteristics of each collection sites are shown in Table 1. Among them, the aridity index was calculated by the ratio of regional precipitation to potential evapotranspiration. The collected seeds were dried in the laboratory and stored in envelope bags at −4°C. In the spring of 2022, seeds were germinated, and seedlings that had reached 6 months of age, characterized by the presence of three tillers, were selected for the subsequent experiment.

**TABLE 1:** The latitude and longitude of nine populations sampled and the major climate variables of *S. krylovii* habitats.

Region	Population code	Latitude (°N)	Longitude (°E)	Annual mean temperature (°C)	Annual mean precipitation (mm)	Aridity index
Eastern region	E1	48.82	119.23	−0.93	324	0.32
	E2	48.55	119.12	−1.25	319	0.31
	E3	48.46	119.04	−1.09	309	0.30
Middle region	M1	43.63	116.68	1.71	317	0.27
	M2	43.60	116.66	1.80	314	0.26
	M3	43.82	116.52	1.93	297	0.24
Western region	W1	43.94	115.86	2.38	263	0.20
	W2	43.91	115.30	1.73	245	0.19
	W3	43.98	115.14	1.46	239	0.18

### 2.2 Experimental Design

A two‐factor (seed source of region × soil moisture treatment) experiment was carried out at Nankai University (Tianjin, China). The experiment was established in plastic pots (14.5 cm in diameter and 12.5 cm in height), with a mixture of vermiculite and nutrient soil (1:1) as culture substrate. Seed source of region included the eastern, middle, and western regions of *S. krylovii* distribution area, with three populations per region (Table 1). Soil moisture treatment was quantified by volumetric water content (VWC) which was estimated by an ECH_2_O Check (Decagon Devices, Pullman, WA, USA). Considering the water retention of nutrient soil used in this study and the corresponding soil water potential in their local habitats in the Inner Mongolia Steppe, we set VWC to 20% for control treatment, 15% for light‐drought treatment, and 8% for heavy‐drought treatment. Each treatment included 18 pots (6 pots per population), each pot included four 6‐month healthy seedlings, and each seedling was similar in size (about 10 tillers, uniform height (10 cm), and root length (12 cm)). In total, there were 162 pots (3 seed source of regions × 3 soil moisture treatments × 18 replications). To exclude precipitation, all pots were randomly placed under a 5‐meter‐height rain‐proof shed, and their positions were changed every week to avoid position effects. The experiment lasted 72 days from July 4, to September 14, 2022.

### 2.3 Data Collection

#### 2.3.1 RNA‐Seq‐Based Transcriptome Data

After 60‐day treatment (September 3, 2022), new full‐expanded leaves were collected and flash frozen in liquid nitrogen, with three replications per treatment. These frozen leaves were stored at −80°C and sent to Shanghai MajorBio Technology Co. Ltd. for RNA‐Seq‐based transcriptome analysis with the standard procedure. Total RNA was extracted using the TRIzol reagent (Invitrogen, USA), and RNA quality was determined by 5300 Bioanalyser (Agilent, USA) and quantified using the NanoDrop 2000 (Thermo Fisher Scientific, USA). One microgram of high‐quality RNA per sample was utilized for library preparation and sequencing, and the sequencing library was performed on NovaSeq X Plus platform using NovaSeq Reagent Kit (Illumina, USA). The RNA‐seq data have been assigned NCBI (National Center for Biotechnology Information) accession PRJNA1154990.

#### 2.3.2 Growth and Biomass

At the end of the experiment (September 14, 2022), the number of tillers was recorded and the plant height was measured as the distance from the highest point of the stem and leaves to the ground for each *S. krylovii* individual. The shoots and roots were carefully harvested by pot and dried in an oven at 60°C for 72  h to obtain the aboveground biomass and belowground biomass.

#### 2.3.3 Physiological Characteristics

After 61‐day treatment, the physiological characteristics of fully expanded leaves, including net photosynthetic rate (Pn, μmol CO_2_ m^−2^ s^−1^), transpiration rate (Tr, mmol H_2_O m^−2^ s^−1^), and stomatal conductance (Gs, mol H_2_O m^−2^ s^−1^), were measured using a Li‐6800 portable photosynthesis measurement system under saturating light conditions of 1300 μmol photons m^2^ s^−1^ (Li‐Cor Biosciences, Lincoln, NE, USA) during 9:00–11:00 a.m. in the morning, five replications per treatment per population. During measurements, the leaf temperature was controlled at 25°C, the relative humidity condition was controlled at 60%, and the ambient CO_2_ concentration was set as 400 μmol CO_2_ mol^−1^.

### 2.4 Data Analysis

#### 2.4.1 Growth and Physiological Traits

General linear models (GLMs) were used to assess the effects of seed source of region, soil moisture treatment, and their interaction on growth traits (the number of tillers, plant height, aboveground biomass, and belowground biomass) and physiological traits (Pn, Tr, and Gs) in SPSS version 27.0 (IBM, USA). When the main factor effect (seed source of region and soil moisture treatment) is significant on a trait, the difference in significance of the trait mean values among the main factor treatments was analyzed by Duncan's multiple comparisons. When the seed source of region × soil moisture treatment interactive effect is significant on a trait, pairwise comparisons of different levels of one factor for fixed levels of the other factors (sometimes called simple main effects) were analyzed by Duncan's multiple comparison.

#### 2.4.2 RNA‐Seq Reads, Preprocessing, and Alignment

A reference genome is not available for *S. krylovii* and a de novo analysis is conducted. Firstly, the raw paired‐end reads are subjected to quality control to remove reads containing adapters and low‐quality reads, resulting in clean reads (Chen et al. 2018). Secondly, high‐quality clean reads filtered out are assembled into transcript sequences using software Trinity (Grabherr et al. 2011). Trinity is a method for efficiently and robustly assembling transcripts from RNA‐seq data without a reference genome. By assembling into unique sequences, further clustering, and scoring, the optimal reference transcript set is constructed. To increase the assembly quality, all the assembled sequences were filtered by CD‐Hit. To obtain the number of reads compared to the assembled transcripts which were used for subsequent expression analysis, the clean reads of each sample were compared with the reference sequence obtained from the Trinity assembly using Bowtie2. Thirdly, the expression levels of the transcripts are quantified to determine their relative abundance in the transcriptome for the subsequent analysis. Fourthly, assembled transcript sequences are compared with databases (such as KEGG and Gene Ontology [GO]) for gene annotation to infer the function of the transcripts.

#### 2.4.3 Transcriptome Analysis

For gene expressions of individuals from the same region, drought adaptation and response mechanisms were analyzed. First, in order to display the effect of treatment on different seed source regions and soil moisture treatment, a PCA analysis was carried out with “prcomp” from R and ggplot2 (Wickham 2009). Second, Venn's diagram of the cross‐soil moisture treatment comparison of gene expressions based on RNA‐seq data from each region identified through using Venny 2.0 (Oliveros 2015). Third, KEGG functional enrichment analysis was carried out based on gene expressions obtained for each treatment (seed source of region × soil moisture treatment) using KEGG Orthology Based Annotation System software (KOBAS, v2.0) in the KEGG pathways. Significant pathways were identified with a cut‐off of corrected *p*‐value lower than 0.05 (Mao et al. 2005). Fourth, a heatmap of intersample correlation was generated from the variance stabilized and transformed counts based on the gene expressions of each treatment using pheatmap R package (Kolde 2019). Fifth, DEGs between drought‐treated seedlings compared to the nondrought control (light‐drought treatment vs. control, heavy‐drought treatment vs. control) were identified (|log_2_FC| ≥ 1 and FDR < 0.05) using the DESeq2 (Love, Huber, and Anders 2014) for individuals from the same region, and the gene expressions level of each DEG was calculated according to the transcripts per million reads (TPM) method using RSEM (Li and Dewey 2011). Venn's diagram of the cross‐seed source of region comparison of DEGs was identified through using Venny 2.0 (Oliveros 2015), obtaining specific DEGs for each region. Volcano plots of DEGs from three regions between heavy‐drought treatment versus control were carried out by the “EnhancedVolcano” R package using default options/parameters. KEGG functional enrichment analysis was carried out based on the DEGs between heavy‐drought treatment versus control for each region.

To explore the drought response mechanisms of *S. krylovii* , KEGG functional annotation analysis was carried out for DCGs between heavy‐drought treatment versus control within the three regions. GO functional enrichment analysis was carried out based on the DCGs between drought treatments versus control within the three regions.

## 3 Results

### 3.1 Growth and Physiological Traits

The factor of seed source had significant effects (*p* < 0.05) on the number of tillers, plant height, aboveground biomass, belowground biomass, and net photosynthetic rate (Pn). These traits obtained from individuals in the western region were significantly (*p* < 0.05) lower than those obtained from individuals in the eastern or middle regions. For example, plant height of individuals in the western region was 19.207 cm, which was significantly (*p* < 0.05) lower than the value of individuals in the eastern region (22.484 cm) and the middle region (21.760 cm) (Table 2).

**TABLE 2:** Effects of seed source (SS), soil moisture (SM), and their interaction on *S. krylovii* traits.

Traits	Seed source of region (SS)	Soil moisture treatment (SM)			Mean ± SE	Results of two‐way ANOVA		
		Control	Light drought	Heavy drought		SS	SM	SS × SM
Number of tillers	The eastern region	21.181 ± 1.132	17.389 ± 1.035	13.889 ± 1.217	17.486 ± 0.680^a^	*F* = 6.017 ***p* = 0.002**	*F* = 22.936 ***p* < 0.001**	*F* = 0.623 *p* = 0.659
	The middle region	19.111 ± 1.111	17.486 ± 1.167	12.278 ± 1.046	16.292 ± 0.667^ab^			
	The western region	16.042 ± 1.389	15.389 ± 1.196	11.167 ± 1.189	14.199 ± 0.740^b^			
	Mean ± SE	18.778 ± 0.714^α^	16.755 ± 0.655^β^	12.444 ± 0.667^γ^				
Plant height (cm)	The eastern region	25.281 ± 0.550	24.560 ± 0.878	17.611 ± 1.081	22.484 ± 0.550^a^	*F* = 8.609 ***p* < 0.001**	*F* = 44.316 ***p* < 0.001**	*F* = 0.424 *p* = 0.792
	The middle region	23.885 ± 0.767	23.249 ± 0.919	18.144 ± 1.050	21.760 ± 0.556^a^			
	The western region	22.253 ± 1.253	21.095 ± 1.179	14.273 ± 1.248	19.207 ± 0.745^b^			
	Mean ± SE	23.813 ± 0.525^α^	22.977 ± 0.582^α^	16.687 ± 0.659^β^				
Aboveground biomass (g)	The eastern region	0.646 ± 0.049	0.641 ± 0.049	0.386 ± 0.045	0.558 ± 0.028^a^	*F* = 4.345 ***p* = 0.013**	*F* = 27.800 ***p* < 0.001**	*F* = 0.344 *p* = 0.848
	The middle region	0.528 ± 0.044	0.562 ± 0.048	0.294 ± 0.027	0.461 ± 0.025^ab^			
	The western region	0.573 ± 0.057	0.500 ± 0.052	0.299 ± 0.050	0.458 ± 0.032^b^			
	Mean ± SE	0.582 ± 0.029^α^	0.568 ± 0.029^α^	0.327 ± 0.024^β^				
Belowground biomass (g)	The eastern region	0.760 ± 0.069	0.594 ± 0.067	0.334 ± 0.038	0.563 ± 0.037^a^	*F* = 3.992 ***p* = 0.019**	*F* = 52.003 ***p* < 0.001**	*F* = 0.313 *p* = 0.869
	The middle region	0.788 ± 0.073	0.579 ± 0.055	0.256 ± 0.030	0.541 ± 0.036^ab^			
	The western region	0.630 ± 0.072	0.503 ± 0.056	0.187 ± 0.029	0.440 ± 0.035^b^			
	Mean ± SE	0.726 ± 0.041^α^	0.559 ± 0.034^β^	0.259 ± 0.019^γ^				
Net photosynthetic rate, Pn (μmol CO_2_ m^−2^ s^−1^)	The eastern region	9.071 ± 1.255^bα^	6.973 ± 1.001b^αβ^	5.092 ± 0.665^bβ^	7.045 ± 0.626^b^	*F* = 21.531 ***p* < 0.001**	*F* = 33.778 ***p* < 0.001**	*F* = 12.329 ***p* < 0.001**
	The middle region	9.854 ± 0.795^bα^	12.157 ± 0.860^bα^	2.516 ± 0.474^cβ^	8.176 ± 0.806^b^			
	The western region	18.789 ± 1.622^aα^	8.545 ± 1.169^aβ^	9.465 ± 0.919^aβ^	12.266 ± 1.058^a^			
	Mean ± SE	12.571 ± 1.031^α^	9.225 ± 0.679^β^	5.691 ± 0.627^γ^				
Transpiration rate, Tr (mmol H_2_O m^−2^ s^−1^)	The eastern region	2.009 ± 0.382	1.475 ± 0.182^b^	1.460 ± 0.208^a^	1.648 ± 0.164	*F* = 0.472 *p* = 0.625	*F* = 15.925 ***p* < 0.001**	*F* = 8.276 ***p* < 0.001**
	The middle region	1.973 ± 0.190^β^	2.614 ± 0.203^aα^	0.389 ± 0.102^bγ^	1.651 ± 0.187			
	The western region	3.188 ± 0.553^α^	1.076 ± 0.291^bβ^	1.288 ± 0.257^aβ^	1.850 ± 0.269			
	Mean ± SE	2.393 ± 0.238^α^	1.723 ± 0.171^β^	1.050 ± 0.142^γ^				
Stomatal conductance, Gs (mol H_2_O m^−2^ s^−1^)	The eastern region	0.087 ± 0.017^b^	0.063 ± 0.008^b^	0.063 ± 0.010^a^	0.071 ± 0.007	*F* = 0.303 *p* = 0.740	*F* = 19.938 ***p* < 0.001**	*F* = 10.422 ***p* < 0.001**
	The middle region	0.088 ± 0.009^bβ^	0.115 ± 0.010^aα^	0.016 ± 0.004^bγ^	0.073 ± 0.008			
	The western region	0.175 ± 0.026^aα^	0.052 ± 0.013^bβ^	0.063 ± 0.011^aβ^	0.097 ± 0.014			
	Mean ± SE	0.117 ± 0.013^α^	0.077 ± 0.008^β^	0.047 ± 0.006^γ^				

*Note:* Under the same soil moisture treatment, the same lowercase letters indicate no significant difference between seed sources at *p* = 0.05 level; under the same seed source of region, the same Greek letters indicate no significant difference between soil moisture treatments at *p* = 0.05 level. The *p*‐value lower than 0.05 is indicated in bold format.

The factor of soil moisture had significant effects (*p* < 0.001) on all traits, with the values obtained under heavy‐drought treatment being significantly lower than those obtained under control treatment and light‐drought treatment. Taking aboveground biomass as an example, it was 0.327 g under heavy‐drought treatment, which was significantly (*p* < 0.05) lower than that under the control treatment (0.582 g) or light‐drought treatment (0.568 g) (Table 2).

The interaction between seed source and soil moisture had significant effects (*p* < 0.001) on Pn, transpiration rate (Tr), and stomatal conductance (Gs), with value of individuals from the middle region under light‐drought treatment (12.157 μmol CO_2_ m^−2^ s^−1^, 2.614 mmol H_2_O m^−2^ s^−1^, and 0.115 mol H_2_O m^−2^ s^−1^) being significantly higher (*p* < 0.05) than that under control treatment (9.854 μmol CO_2_ m^−2^ s^−1^, 1.973 mmol H_2_O m^−2^ s^−1^, and 0.088 mol H_2_O m^−2^ s^−1^). Under the heavy‐drought treatment, the Pn of individuals from the western region (9.465 μmol CO_2_ m^−2^ s^−1^) was significantly (*p* < 0.05) higher than that from the eastern region (5.092 μmol CO_2_ m^−2^ s^−1^) or the middle region (2.516 μmol CO_2_ m^−2^ s^−1^) (Table 2).

### 3.2 Gene Expressions

#### 3.2.1 Transcriptome Profile Analysis

By PCA, the samples were separated by soil moisture treatment (the horizontal axis) and seed source of region (the vertical axis). Individuals under the heavy‐drought treatment were separated from individuals from the control and light‐drought treatment. Individuals from the eastern and middle regions were clustered together and separated from those from the western region. However, there was relatively lower variance explained due to the relatively larger area of seed source (Figure S1).

#### 3.2.2 KEGG Analysis on Gene Expression

**FIGURE 1:**
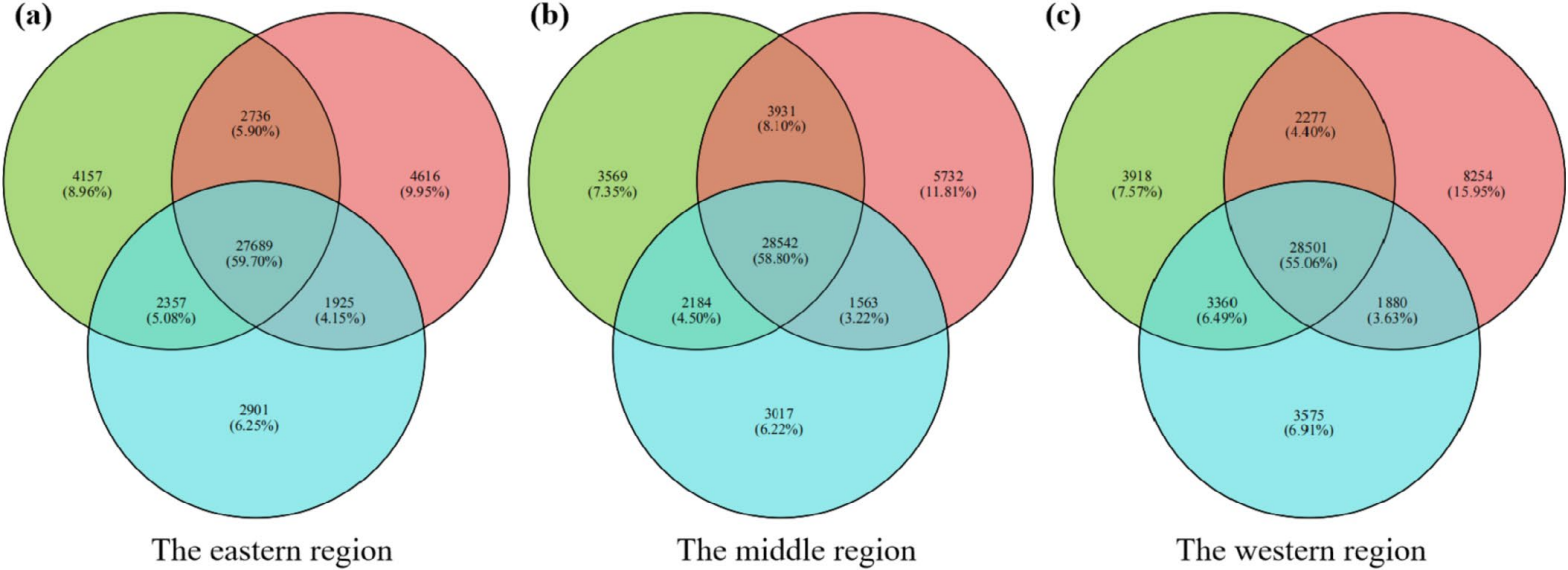
Venn diagrams of expressed genes of *S. krylovii* from the eastern region (a), middle region (b), and western region (c) under control (green), light‐drought treatment (blue), and heavy‐drought treatment (red).

**FIGURE 2:**
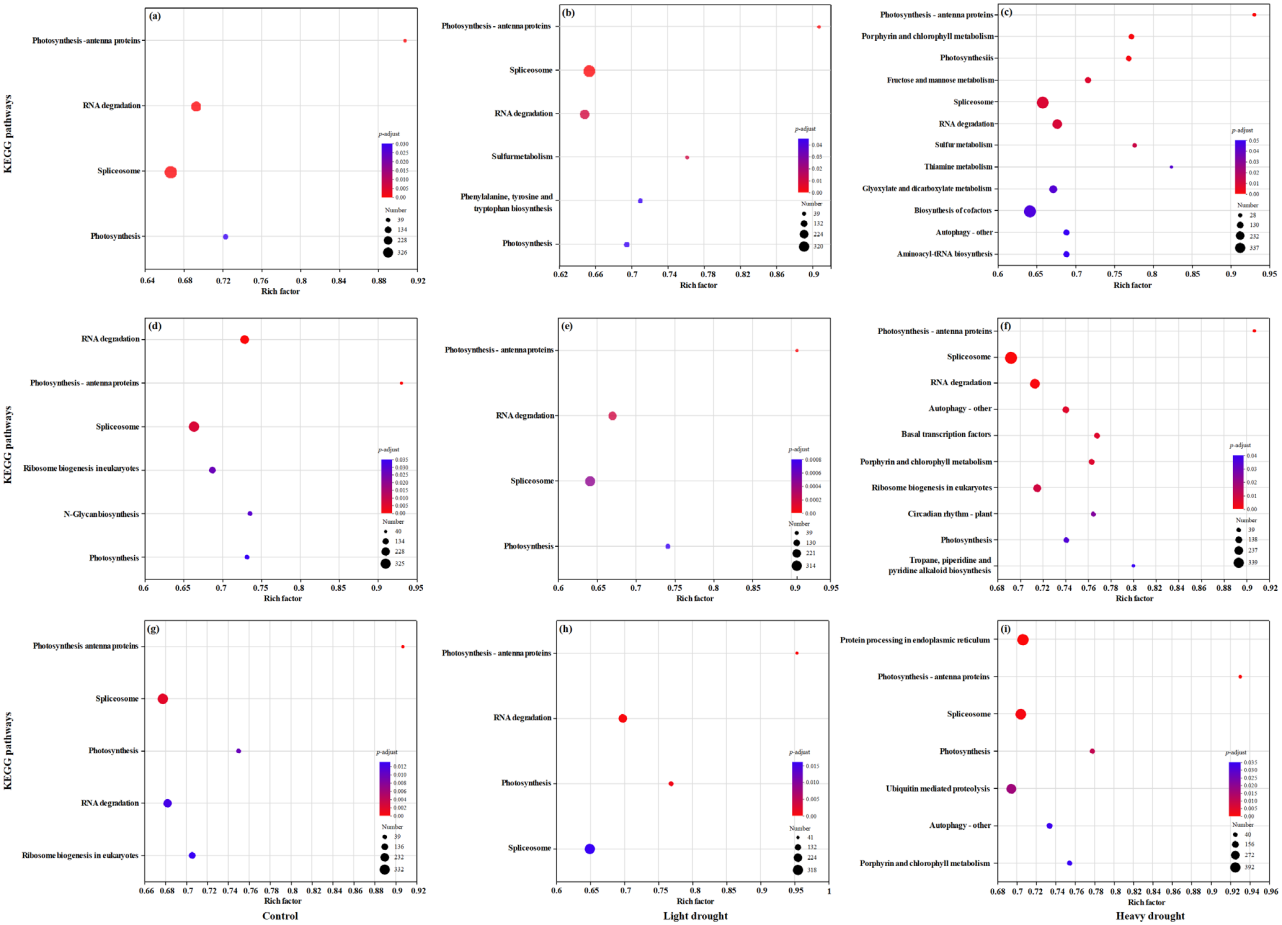
KEGG functional enrichment analysis of gene expressions in the eastern region (a–c), middle region (d–f), and western region (g–i) under three soil moisture treatments.

The gene expressions and KEGG functional enrichment results for each region under different soil moisture treatments are shown in Figures 1 and 2. For individuals from the eastern region, gene expressions were significantly enriched in photosynthesis antenna proteins, RNA degradation, spliceosome, and photosynthesis (Figure 2a–c). Specifically, gene expressions were enriched in sulfur metabolism pathway and phenylalanine, tyrosine, and tryptophan biosynthesis pathway under the light‐drought treatment (Figure 2b), and in porphyrin and chlorophyll metabolism, thiamine metabolism, and fructose and mannose metabolism pathways under the heavy‐drought treatment (Figure 2c).

For individuals from the middle region, gene expressions were mainly enriched in photosynthesis antenna proteins, RNA degradation, spliceosome, and photosynthesis (Figure 2d–f). Specifically, gene expressions were enriched in basal transcription factors, circadian rhythm plant, and ribosome biosynthesis in eukaryote pathways under the heavy‐drought treatment (Figure 2f).

For individuals from the western region, gene expressions were mainly enriched in photosynthesis antenna proteins, spliceosome, and photosynthesis (Figure 2g–i). Specifically, gene expressions were enriched in RNA degradation under the light‐drought treatment (Figure 2h), and in protein processing in endoplasmic reticulum and ubiquitin‐mediated proteolysis pathways under the heavy‐drought treatment (Figure 2i).

**FIGURE 3:**
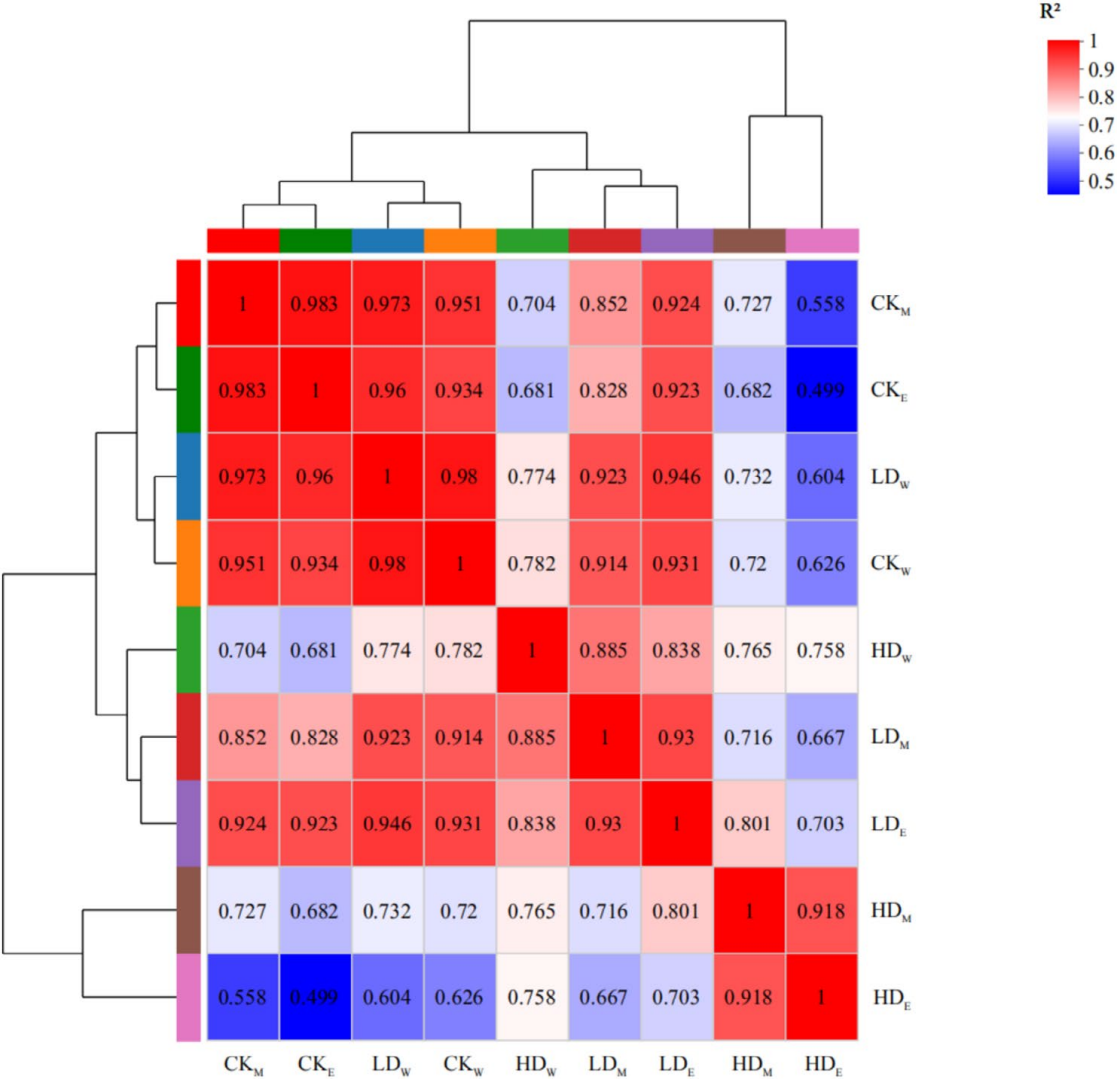
Correlation clustering heat map of gene expression level of *S. krylovii* from the eastern region (E), middle region (M), and western region (W) under control (CK), light‐drought (LD), and heavy‐drought (HD) treatments.

Results of heatmaps and the dendrogram showed that individuals from the eastern and middle regions showed higher similarity and grouped into one subgroup under the same soil moisture treatments. Individuals from the eastern region under the heavy‐drought treatment showed higher similarity with those from two other regions under the light‐drought treatment. Individuals from the western region under both control and light‐drought treatments showed higher similarity, and this subgroup was clustered with another subgroup that included the eastern and middle regions under the control treatment (Figure 3).

### 3.3 DEGs and Differentially Coexpressed Genes (DCGs)

#### 3.3.1 Distribution of DEGs and DCGs

**FIGURE 4:**
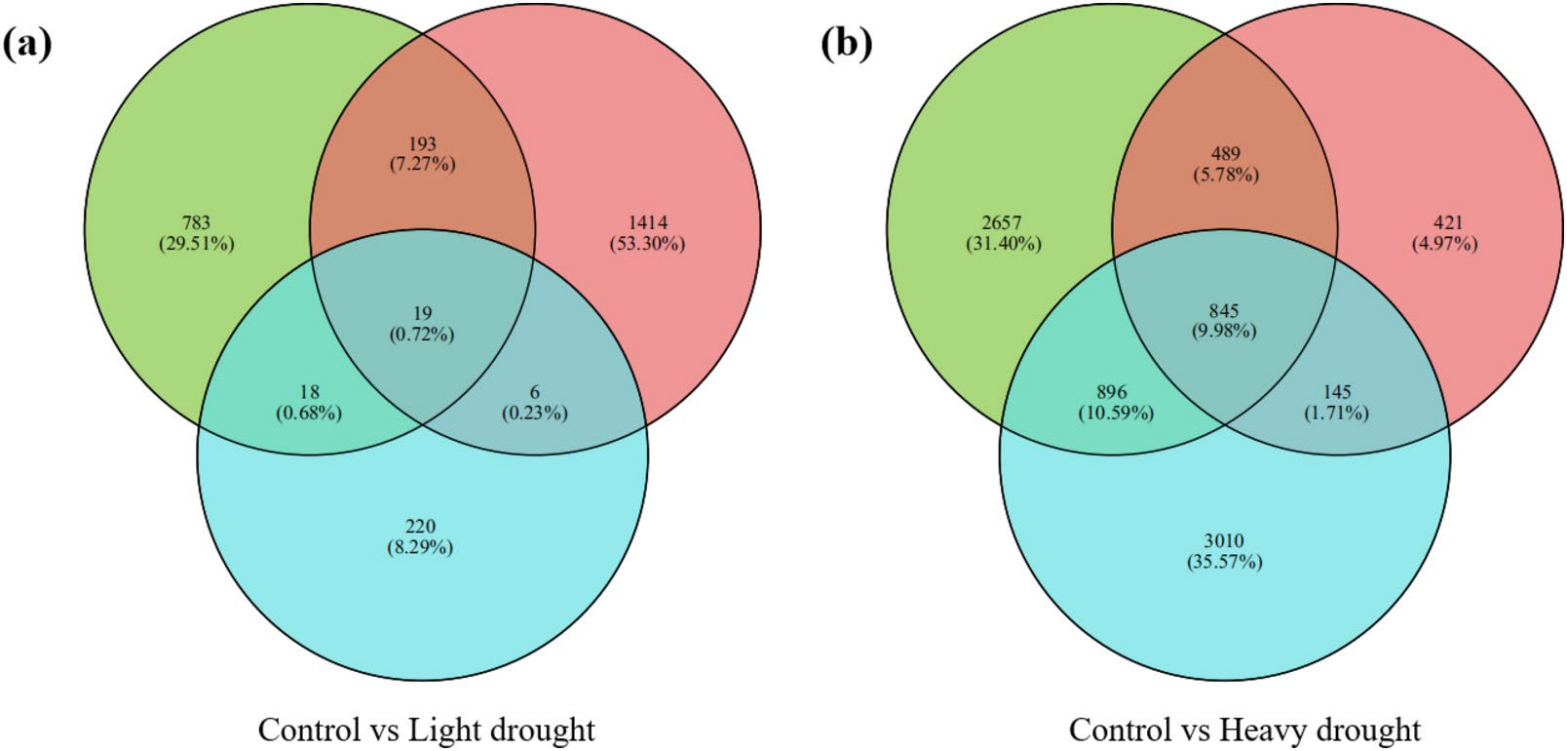
Venn diagram showing the distribution of differentially expressed genes (DEGs) in *S. krylovii* from the eastern region (green), middle region (red), and western region (blue) between light‐drought treatment versus control (a) and heavy‐drought treatment versus control (b).

**FIGURE 5:**
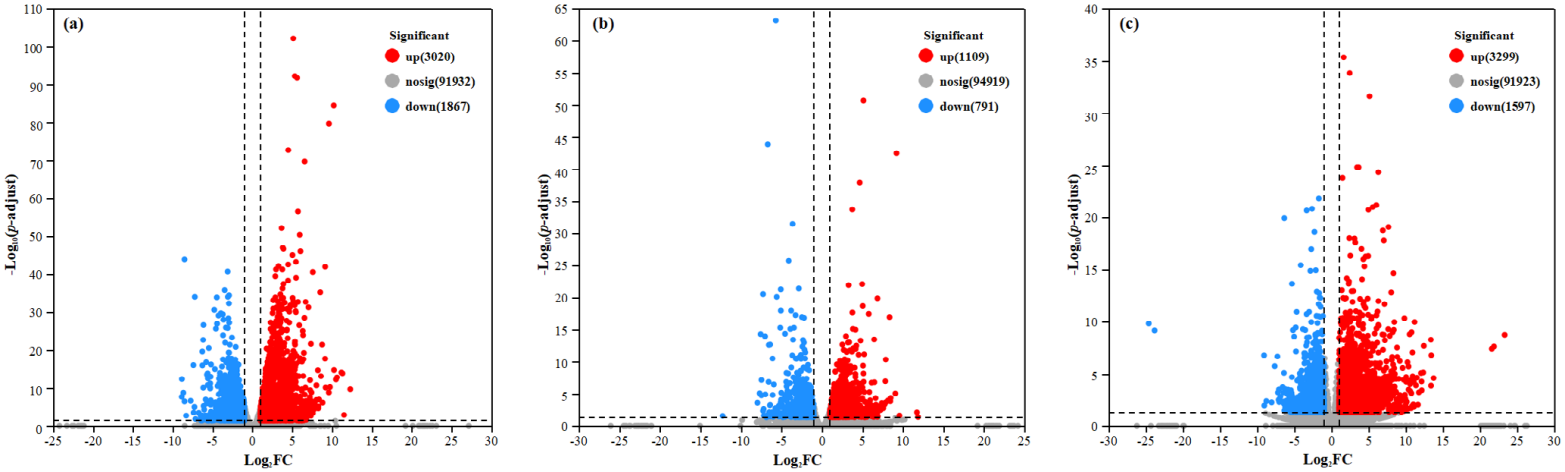
Volcano plots of DEGs of *S. krylovii* from the eastern region (a), middle region (b), and western region (c) between heavy‐drought treatment versus control.

A significant number of genes were found to be upregulated or downregulated under heavy‐drought treatment compared to the control and light‐drought treatments. The DEGs between drought treatments versus control treatment showed region‐specific and treatment differences, with greater DEGs (4887 from the eastern region with 3020 genes upregulated and 1867 downregulated; 1900 from the middle region with 1109 genes upregulated and 791 genes downregulated; and 4896 from the western region with 3299 genes upregulated and 1597 genes downregulated) between heavy‐drought versus control treatment than that between light‐drought versus control treatment (1013 from the eastern region; 1632 from the middle region; and 263 from the western region) (Figures 4 and 5).

In addition, the DCGs within these three regions were 19 (0.72%) between light‐drought treatment versus control treatment (Figure 4a), with 9 being upregulated and 10 being downregulated (Table S1); and 845 (9.98%) between heavy‐drought treatment versus control treatment (Figure 4b), with 592 being upregulated and 253 being downregulated (Table S2). Therefore, in this study, we further analyzed DEGs and DCGs under heavy‐drought compared to the control treatment by KEGG functional annotation analysis and GO functional enrichment analysis.

#### 3.3.2 KEGG Pathway Analysis on DEGs Between Heavy‐Drought Versus Control Treatment

**FIGURE 6:**
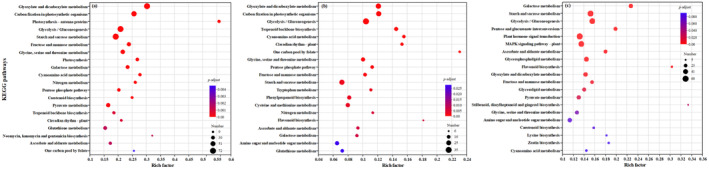
KEGG functional enrichment analysis of differentially expressed genes (DEGs) in *S. krylovii* from the eastern region (a), middle region (b), and western region (c) between heavy‐drought treatment versus control.

For individuals from the eastern region, DEGs were mainly enriched in the pathways of photosynthesis antenna proteins (with 24 genes upregulated), cyanoamino acid metabolism (with 13 genes upregulated and 12 genes downregulated), and photosynthesis (with 28 genes upregulated and 1 gene downregulated) for individuals from the eastern region (Figure 6a; Table S3). For individuals from middle region, DEGs were mainly enriched in the pathways of one carbon pool by folate (with 7 genes upregulated and 2 genes downregulated), cyanoamino acid metabolism (with 9 genes upregulated and 5 genes downregulated), and carbon fixation in photosynthetic organisms (with 27 genes upregulated) for individuals from middle region (Figure 6b and Table S4). For individuals from the western region, DEGs were mainly enriched in the pathways of flavonoid biosynthesis (with 7 genes upregulated and 3 genes downregulated), galactose metabolism (with 25 genes upregulated and 7 genes downregulated), and glycolysis/gluconeogenesis (with 39 genes upregulated and 13 genes downregulated) for individuals from the western region (Figure 6c and Table S5). No significant pathway of DEGs between light‐drought treatment versus control treatment was enriched in individuals from the middle and western regions.

#### 3.3.3 KEGG and GO Analysis on DCGs Between Heavy‐Drought Versus Control Treatment

**FIGURE 7:**
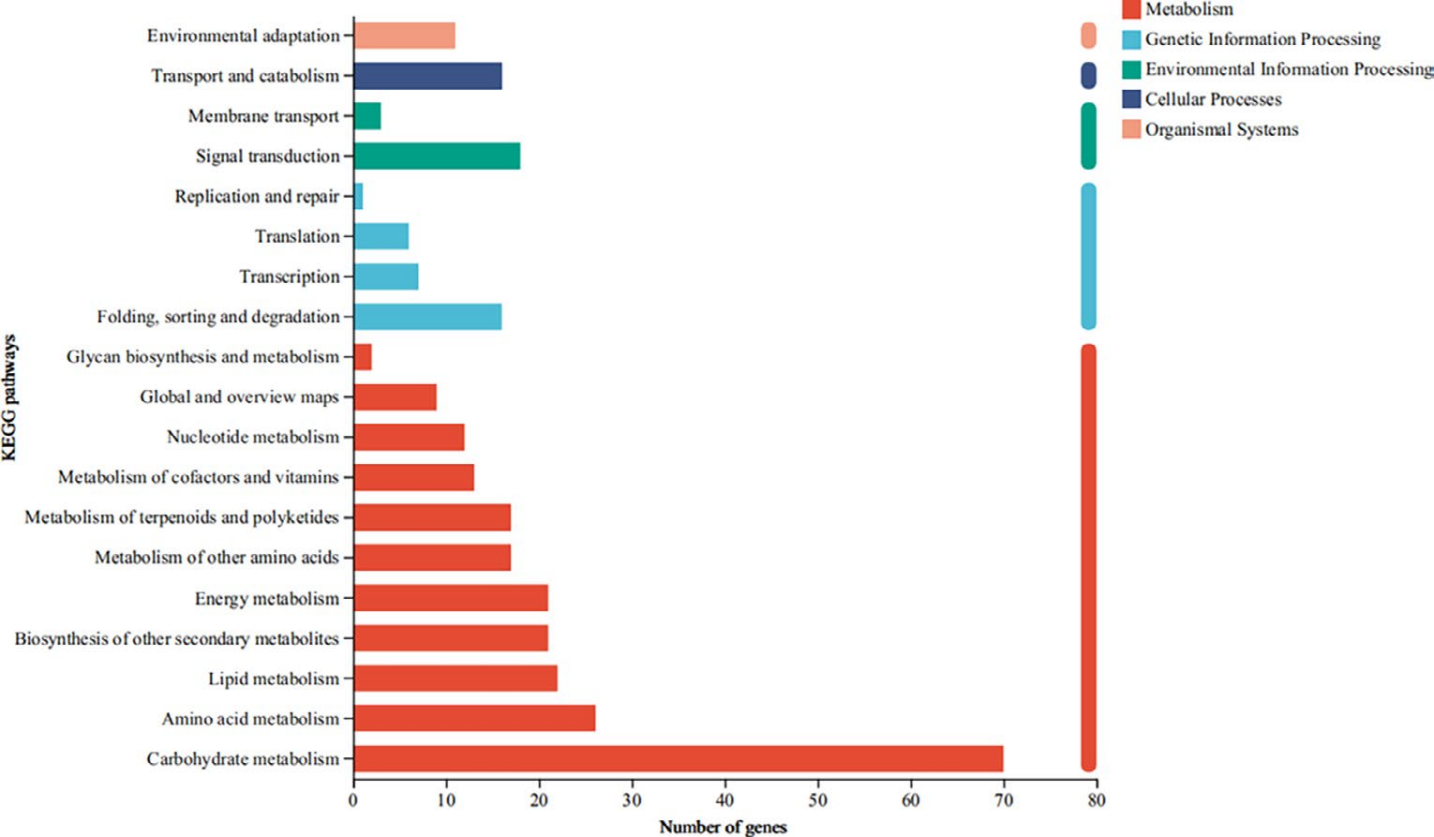
Differentially coexpressed genes (DCGs) and KEGG functional annotation analysis of *S. krylovii* from three regions under heavy‐drought treatment versus control.

By KEGG functional annotation analysis on 845 DCGs, biochemical metabolic pathways of the first category were identified: metabolism, genetic information processing, environmental information processing, cellular processes, and organismal systems. The pathways of second category were enriched, such as carbohydrate metabolism, amino acid metabolism, and lipid metabolism (Figure 7). Among them, a large number of genes were enriched in the pathways whose descriptions were glycolysis/gluconeogenesis (with 18 genes upregulated and 2 genes downregulated), starch and sucrose metabolism (with 13 genes upregulated and 3 genes downregulated), and MAPK signaling pathway—plant (with 7 genes upregulated and 4 genes downregulated) and plant hormone signal transduction (with 7 genes upregulated and 3 genes downregulated) (Tables S6 and S7).

By GO functional enrichment analysis on 845 DCGs, most of genes were enriched in the pathways whose descriptions were amide transport (with 4 genes upregulated and 4 genes downregulated), rhythmic process (with 6 genes upregulated and 4 genes downregulated), and pyruvate metabolic process (with 9 genes upregulated and 7 genes downregulated) (Table S2).

## 4 Discussion

### 4.1 Drought Adaptation Differences of *S. krylovii* Among Different Regions

In this study, the factor of seed source of region showed significant effects on growth and physiological traits (Table 2), which was consistent with the previous studies (Klein and Mitchell 2024). In addition, under the heavy‐drought treatment, unique enriched pathways were found in individuals from each region.

For individuals from the eastern region under heavy‐drought treatment, specific KEGG‐enriched pathways included thiamine metabolism, fructose, and mannose metabolism (Figure 2c), and these metabolism pathways are found to be related to drought tolerance in several researches. Li et al. (2022) have shown that exogenous application of thiamine can enhance plant stress tolerance by activating stress‐responsive genes and calcium signal transduction. Ye et al. (2020) have found that *Pterocarya stenoptera* initiates the thiamine metabolism pathway under drier air stress conditions using 6‐month‐old seedlings. You et al. (2019) have shown that genes for fructose and mannose metabolism are upregulated in drought‐tolerant sesame to cope with stress conditions.

For the individuals from the middle region under heavy‐drought treatment, specific KEGG‐enriched pathways included basal transcription factors and circadian rhythm–plant (Figure 2f). Circadian rhythm–plant pathway is common for species under the drought condition (Wang et al. 2021). Transcription factors pathway is an important class of regulatory proteins that plays important roles in regulatory networks and signaling pathways of plant development and abiotic stresses (Yang et al. 2017); however, the basal transcription factors pathway related to drought adaptation was not common in previous studies. Therefore, more specific researches on transcription factor pathways are needed for *S. krylovii* in the future.

For the individuals from the western region under heavy‐drought treatment, specific KEGG‐enriched pathways included protein processing in endoplasmic reticulum, ubiquitin‐mediated proteolysis (Figure 2i). This finding was consistent with the previous studies. Moon et al. (2018) have found that the drought tolerance mechanisms of the drought‐tolerant potato ( *Solanum tuberosum* L.) are related to protein processing in the endoplasmic reticulum as well as the photosynthetic antenna protein. Zhang et al. (2021) have shown that endoplasmic reticulum and capacity of ubiquitin‐mediated proteolysis pathways play important roles in gene expression of Bt cotton under high‐temperature and water‐deficit stress conditions.

### 4.2 Drought Response Differences of *S. krylovii* Among Different Regions

Plant drought responses have been proven highly variable and significantly associated with higher environmental heterogeneity (Akman, Carlson, and Latimer 2021). In this study, the diagram based on gene expressions for all treatments also showed that individuals from eastern and middle regions under the same soil moisture treatment showed more similarity (Figure 3), as shown by physiological traits in Table 2. In addition, under heavy‐drought treatment, the decline rates of the number of tillers in the western region were much smaller and the Pn of individuals from the western region were significantly greater than that of the other two regions (Table 2). Furthermore, under heavy‐drought treatment, the KEGG‐enriched pathways in the individuals from the western region were less than in the other two regions, which indicated that individuals from the western region were tolerant while individuals in the other two regions were sensitive to heavy‐drought treatment (Guo et al. 2023). All these findings indicated that the greater drought tolerance of individuals from the western region could explain the eastern shifts of the distribution areas of *S. krylovii* to some extent (Liu 2004).

Based on KEGG on DEGs between heavy‐drought treatment versus control, the present study showed that there were different response strategies between individuals from the western region and those from the other two regions. For DEGs of RNA‐seq data, both upregulated and downregulated genes are important for species responses to stress conditions. However, sorting downregulated genes into tolerance and response strategies is difficult due to the fact that many cellular processes are attenuated under stress conditions (such as heavy‐drought stress) to reduce growth and initiate dormancy (Bushman et al. 2021). Therefore, most studies have paid more attention to the significantly enriched pathways of DEGs.

First, metabolism of other amino acids and energy metabolism were significantly enriched and upregulated in individuals from the eastern and middle regions but not the western region (Figure 6a,b, Tables S3 and S4). Many studies have shown that drought stress leads to changes in amino acid content and energy metabolism in plants (Heinemann et al. 2021; Ünlüsoy et al. 2023). Amino acid accumulation has a twofold role, their availability for protein biosynthesis and accelerated recovery after stress and osmoprotectant activity (Ashrafi et al. 2018). Wan et al. (2021) have found that differentially expressed metabolites related to the acid metabolism pathways may be key factors affecting drought resistance differences in two cherry (*Prunus pseudocerasus* L.) rootstocks. Plants can accumulate soluble proteins to maintain cell expansion and cell membrane stability, thereby protecting macromolecules from damage under drought stress (Fang and Xiong 2015). Zhu et al. (2017) have found that individuals of *Sorghum sudanense* were significantly enriched in photosynthesis (energy metabolism) by KEGG on DEGs between drought treatment versus control. Plant photosynthesis can directly affect plant tissue material production and is extremely sensitive to environmental changes, thus, drought stress has a great impact on plant photosynthesis (Basu et al. 2016).

Second, carbohydrate metabolism and biosynthesis of other secondary metabolites were significantly enriched and upregulated in individuals from the western region (Figure 6c and Table S5). In response to drought stress, an increase in the content of flavonoids can help eliminate excess reactive oxygen species in the plant and help the plant to better adapt to the conditions of drought stress (Nakabayashi, Mori, and Saito 2014). Many studies have shown that drought stress usually induces the accumulation of flavonoids in plants (Gao et al. 2021; Yu et al. 2022). Glycolysis is an important metabolic pathway in carbohydrate metabolism, and changes in glycolysis and gluconeogenesis are considered an essential feature of plant adaptation to abiotic stresses (Broeckling et al. 2005). Shi et al. (2020) have conducted transcriptomic analysis of okra ( *Abelmoschus esculentus* ) under drought stress and have shown that most of the DEGs were mainly enriched in carbon metabolism, secondary metabolite synthesis, glycolysis/glycolysis, etc. The increase in soluble sugar content plays an important role as a signaling molecule for plant growth and development under environmental stress and also is involved in the process of cellular carbon and energy metabolism (Rolland, Baena‐Gonzalez, and Sheen 2006; Shahbazy et al. 2020). Using peach (*Salvadora persica*) as the experimental material, Rangani, Panda, and Parida (2020) have found that galactose is upregulated to provide roles such as osmoregulation, energy for antioxidant defense, carbon skeleton for secondary metabolite synthesis, and stress signaling under drought stress.

### 4.3 Drought Responses of *S. krylovii*

When plants are subjected to drought stress, they usually reduce its photosynthetic capacity to reduce water consumption, reduce their growth, and ensure their survival (Dias et al. 2007; Poorter et al. 2012), and similar results were found in this study (Table 2). Furthermore, we tried to explore the functioning changing of *S. krylovii* facing to heavy‐drought treatment based on RNA‐seq data. The significantly enriched pathways by KEGG based on DCGs were glycolysis/glycoconeogenesis, starch and sucrose metabolism, and MAPK signaling pathway—plant and plant hormone signal transduction, with relative larger number of genes significantly upregulated (Tables S6 and S7). This finding indicated that *S. krylovii* compensated for the damage under the drought stress condition by continuously enhancing its metabolic vigor, which was consistent with the drought stress on Kentucky bluegrass ( *Poa pratensis* ) (Bushman et al. 2021). Similar pathways of responses to drought stress were found in other species, such as glycolysis/glycoconeogenesis pathway for okra ( *Abelmoschus esculentus* ) (Shi et al. 2020), starch and sucrose metabolism pathway for *Verbena bonariensis* (Wang et al. 2018), plant hormone signal transduction pathway for foxtail millet ( *Setaria italica* ) (Qin et al. 2020), and MAPK signaling pathway—plant for peanut ( *Arachis hypogaea* ) (Zhao et al. 2021).

## 5 Conclusions

This study shows that the drought adaptation and response mechanisms of *S. krylovii* are related to their seed source of regions, with significantly different mechanisms for individuals from the western region and those from the other two regions. In addition, there is relatively higher drought adaptability and tolerance for individuals from western region. In the area of grassland vegetation in the Inner Mongolia Steppe, increasing aridity would cause a significant reduction in grassland regions, but the distribution area of *S. krylovii* increased. The present findings could provide some explanations for the distribution areas increase and eastern shifts of *S. krylovii* , and could help us understand and predict the evolution potential of this important species.

## Author Contributions

**Ziqing Gong:** conceptualization (equal), formal analysis (equal), methodology (equal), software (equal), writing – original draft (lead). **Zehang Qu:** data curation (equal), methodology (equal), software (equal). **Yulin Liu:** conceptualization (equal), methodology (equal). **Tao Wang:** investigation (equal), software (equal). **Baijie Fan:** formal analysis (equal), methodology (equal). **Anzhi Ren:** supervision (equal), writing – review and editing (equal). **Yubao Gao:** supervision (equal), writing – review and editing (equal). **Nianxi Zhao:** conceptualization (equal), funding acquisition (equal), supervision (equal), writing – review and editing (equal).

## Conflicts of Interest

The authors declare no conflicts of interest.

## Supporting information


**Figure S1.** Principal component analysis of gene counts for *S. krylovii* from the eastern, middle, and western regions under control, light‐drought, and heavy‐drought treatments.

Tables S1–S7.

## Data Availability

The RNA‐seq data have been assigned NCBI accession PRJNA1154990. The data will be archived in Dryad (publicly accessible repository) before the article is accepted.
