# Supplementary material for: Drought Adaptation and Responses of Stipa krylovii Vary Among Different Regions: Evidence From Growth, Physiology, and RNA‐Seq Transcriptome Analysis
Source: Ecol Evol. 2025 Jan 21;15(1):e70870. doi: 10.1002/ece3.70870 (PMC11747353; doi:10.1002/ece3.70870)
Supplement: Supplementary file 1 — Figure S1. Principal component analysis of gene counts for S. krylovii from the eastern, middle, and western regions under control, light‐drought, and heavy‐drought treatments. [file ECE3-15-e70870-s001.docx]

**Supplemental figure for ‘‘Drought adaptation and responses of *Stipa krylovii* vary among different regions: evidence from growth, physiology and RNA-seq transcriptome analysis ”**

**Ziqing Gong, Zehang Qu, Yulin Liu, Tao Wang, Baijie Fan, Anzhi Ren, Yubao Gao, Nianxi Zhao**

**Supplemental Figure**


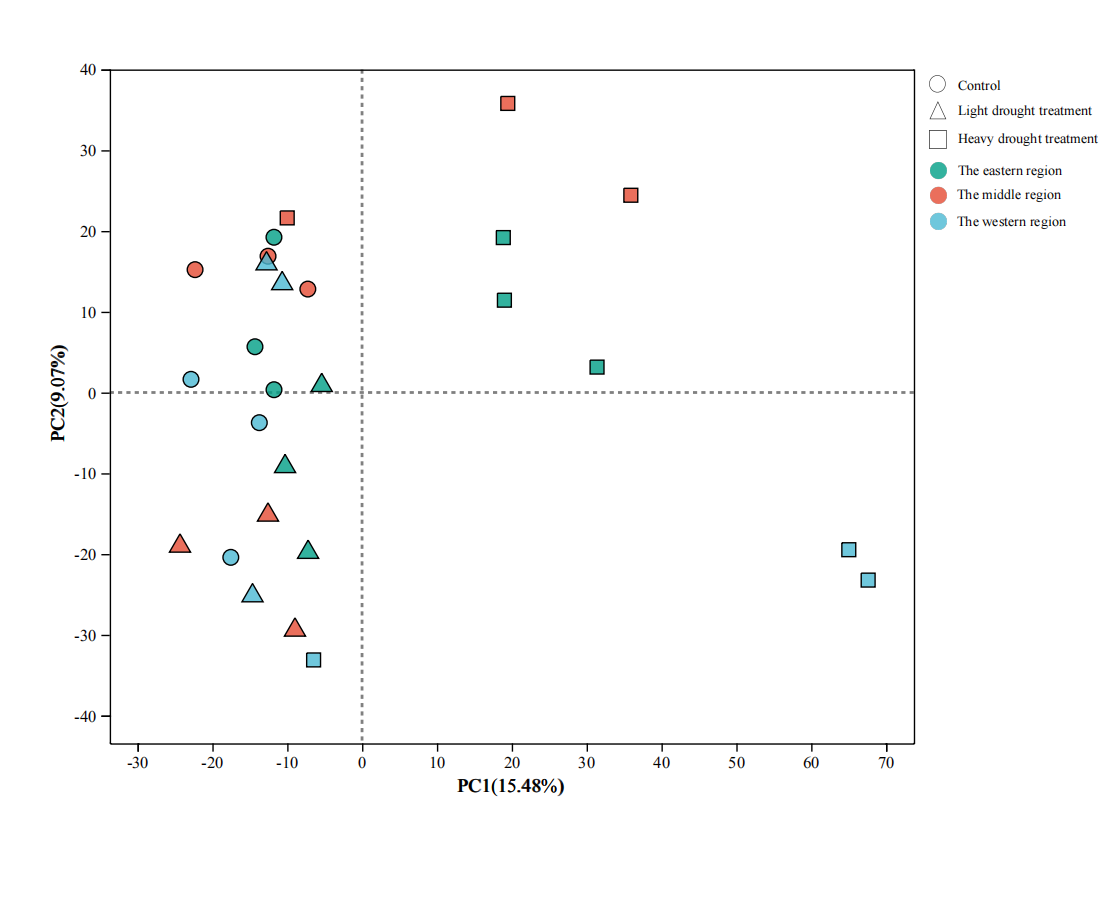


**Fig. S1** Principal component analysis of gene counts for *S. krylovii* from the eastern, middle and western regions under control, light drought and heavy drought treatments.
